# Detection of *Neospora Caninum* DNA in Wild Birds from Italy

**DOI:** 10.3390/pathogens8040202

**Published:** 2019-10-23

**Authors:** Simona Nardoni, Alessandro Poli, Ilaria Varvaro, Guido Rocchigiani, Renato Ceccherelli, Francesca Mancianti

**Affiliations:** 1 Dipartimento di Patologia Animale, Università di Pisa, 56100 Pisa, Italy; alessandro.poli@unipi.it (A.P.); ilaria5787@virgilio.it (I.V.); guido.rocchigiani.g@gmail.com (G.R.); 2 Centro Recupero Uccelli Marini e Acquatici-CRUMA, 57121 Livorno, Italy; apusvet.cruma@libero.it (R.C.); francesca.mancianti@unipi.it (F.M.)

**Keywords:** *Neospora caninum*, wild birds, IFAT, PCR

## Abstract

The role of avian species in the *Neospora caninum* life cycle has not been completely elucidated, and epidemiological data from Europe are scant. The aim of the present report was to evaluate the presence of *N. caninum* DNA in the tissues of 302 birds belonging to different avian species, along with IFAT titers. Forty-two out of the 302 birds (13.9%) showed low serological titers (1/50 and 1/100) against *N. caninum.* These data, positive for 31 animals (10.3%), were corroborated by PCR. Twenty-two hearts and eighteen brains scored positive, while nine subjects resulted had parasite DNA both in their hearts and brains. Serological data showed significantly higher results in waterfowl in respect to non-waterfowl avian species. This finding indicates a higher exposure of water birds to the parasite. These avian species, in fact, which feed directly from soil and/or water, are prone to ingest oocysts excreted by final canid hosts. The present study adds information to the state of art of *N. caninum* epidemiology in Italy, even if more investigations using bio-assays are needed to allow for a serological/parasitological follow-up to evaluate the real impact of the avian species in maintaining the parasite in main reservoirs.

## 1. Introduction

*Neospora caninum* is an Apicomplexan protozoan, strictly related to *Toxoplasma gondii,* misidentified until 1988 [1]. The organism is responsible for neuromuscular disease in dogs, and it is considered a major abortifacient agent in ruminants [2]. In cattle, abortions follow sporadic, endemic, and epidemic patterns. In particular, epidemic abortions storms are devastating and very costly, with a large proportion of at risk cows [3,4].

The parasite has the dog as a final/intermediate host; in intermediate hosts, it may furthermore display a vertical transmission, which is recognized as an important route for parasite dissemination [5]. 

*N. caninum* shows several similarities with *T. gondii* in its life cycle, even if viable parasites have been isolated only from few mammal species [6], and the zoonotic role of the parasite is unascertained because the parasite has never been isolated from human tissues [7].

The role of avian species in the *N. caninum* life cycle has not been completely elucidated. Some serological studies have been performed with different prevalence rates, and experimental infections attempts, microscopical investigations, and molecular investigations have recently been revised [6]. Domestic avian species such as quail and chickens appear resistant to infectious challenge [8,9], and the latter have been shown to be unable to infect dogs following a bioassay. Similarly, a bioassay failed after the administration of experimentally infected doves to dogs [10]. On the other hand, wild avian species have been demonstrated to harbor parasite DNA [11,12,13,14], as well as tissue cysts [15].

Data from Europe are scant and consist of a serological study on common ravens (*Corvus corax*) and serological reports on wild birds from Spain [12,16], followed by a serological and molecular investigation in waterfowl from Italy [14]. 

The aim of the present report was to evaluate the presence of *N. caninum* DNA in tissues of various free ranging birds, along with antibody titers, to shed light on the involvement of different wild avian species in the life cycle of this intriguing parasite. 

## 2. Results

Forty-two out of the 302 birds (13.9%) showed low serological titers (1/50 and 1/100) against *N. caninum.* These data were corroborated by PCR, the results of which were positive for 31 animals (10.3%). Twenty-two hearts scored positive, along with eighteen brains, while nine subjects were shown to have parasite DNA both in their hearts and brains. 

Waterfowl showed a seroprevalence of 17.5% and a PCR positive prevalence of 12.1%, while the other avian species were seropositive at 8.3% and PCR positive at 7.5%. The sequences obtained with high quality were 280–300 bp long, all identical and all sharing 99% of similarity with many other *N. caninum* isolates (Genbank: KP715559; KP715560; LN714476).

The differences in the seroprevalences between waterfowls (*Anas* spp. and *Vanellus vanellus*) and non-waterfowls scored as significant (O.R. 2.35 and *p* = 0.0230).

More detailed data are reported in Table 1.

## 3. Discussion

The studied animals showed an overall seroprevalence of 13.9%, with low antibody titers. Waterfowl, in particular, were more seropositive (17.5%) than other species (8.3%). Data on wild birds from the literature have shown seroprevalences ranging from 0% in Brazil [15] to 35.8% in common ravens in Spain [16]. Data from Italy in a previous study carried out on waterfowls (34.3%) [14] strongly differed from the present results. This finding may be due to different environmental conditions. The 2018/2019 autumn/winter period, in fact, was very dry (low rainfalls accounted for less than 20%), in comparison with the past (unpublished data). Dry weather limits waterfowl migration behavior, so the examined waterfowl could have been non-migratory, resident subjects. Serological data were significantly higher in waterfowl in respect to non-waterfowl avian species. This finding indicates a higher exposure of water birds to the parasite. These avian species, in fact, feed directly from soil and/or water and are therefore prone to ingest oocysts excreted by final canid hosts [5].

PCR studies in wild birds yielded different results, with positive results ranging from 0% in South Africa [17] to 28.6% in waterfowl (*Anas crecca*, *Anas platyrhynchos*, *Anas penelope*, *Anas acuta, V. vanellus*) from Italy [14]. The findings showed to birds from the rescue center (7.5%) are in particular in agreement with the positivity rates (7.5%) found in sparrows from Brazil [11] and in rock pigeons *(Columbia livia)* (9.2%) from Pakistan [18].

The present investigation is the first carried out on a large number of wild birds in Italy, and, to the best of our knowledge, there have been no other molecular studies with antibodies determination. 

As previously reported [14], the choice of two different tissues (brain and heart) for PCR determinations strongly enhances the sensitivity of test. Thirteen animals yielded positivity in heart testing, nine for the brain and nine for both the brain and the heart, indicating the usefulness of the sampling of more than one tissue.

The present study is moreover the first report of *N. caninum* infection in *Columba palumbus* and in *Larus ridibundus.* Scavenger and carnivorous birds have been reported as infected [12,16], and seagulls have scavenging habits. On the other hand, eared doves showed a high seropositivity in Brazil [6], as did rock pigeons in Pakistan [18]. 

## 4. Materials and Methods 

The study was carried out on 302 birds belonging to different avian species for epidemiological purposes, and it was commissioned by the Veterinary Parasitology Lab of the Department of Veterinary Sciences of the University of Pisa. Some of the birds (n = 182) ere waterfowl from regular hunting activity. The other birds (n = 120) came from a rescue center for avian species (CRUMA) and were injured wild animals, dead during hospitalization, or were found dead. All the subjects were from the provinces of Pisa and Leghorn (Tuscany, Central Italy). Detailed data about the species and gender of the subjects are reported in Table 2. 

The carcasses were sent to the lab as soon as possible and were maintained at 4 °C until processed. Whole hearts and brains [11,14], along with intracardiac coagules, were collected from all the subjects for molecular studies to carry out the serological determinations. All tissues were kept at –20 °C until processing. 

Serological test consisted of an IFAT carried out using 12-well slides (Fullerton Lab, Fullerton, California), with a threshold dilution 1/50, as previously reported [14].

DNA was extracted by a QIAamp^®^ DNA mini-kit (Qiagen, Milan, Italy) in accordance with the manufacturer’s instructions. After extraction, DNA was stored at –20 °C until use. PCR was performed as described by Müller et al. [19], using the Np6plus (CTCGCCAGTCAACCTACGTCTTCT) and Np21plus (CCCAGTGCGTCCAATCCTGTAAC) primers that amplify a 337 base pair fragment of the Nc5 region of the genomic DNA. DNA were extracted with the same procedure from the CNS of a dead puppy and histologically diagnosed with neosporosis, showing a *N. caninum* high serological titer; numerous cerebral protozoarian cysts and water were used as positive and negative controls, respectively; a brain from a mouse SPF was extracted and used as a further negative sample. All amplicons obtained were purified using the QIAquick PCR purification kit (Qiagen), according to the manufacturer’s instruction, and then sequenced and analyzed. Sequencing was performed by a commercial laboratory (BMR-Genomics, Padova, Italy). Sequences were assembled by combining both sense and antisense strands, and they were then corrected by a visual analysis of the electropherogram using Bioedit v.7.0.2 before being then compared with those available in GenBank using the BLAST program (http://www.ncbi.nlm.nih.gov/BLAST). 

The differences between the serological and molecular results from waterfowl and non-waterfowl were analyzed by a chi squared test.

## 5. Conclusions

The present study adds information to the state of art of *N. caninum* epidemiology in Italy, confirming the occurrence of both DNA and antibodies in wild avian species. Different birds have been recognized to harbor parasite DNA, suggesting a possible colonization by *N. caninum*. These animals enhance the possibility of canine infection following the ingestion of infected preys. 

All the cases from literature have been transversal studies, so more investigations using bio-assays are needed to allow for a serological/parasitological follow-up to evaluate the real impact of avian species in maintaining the parasite in main reservoirs. 

## Figures and Tables

**Table 1:** Results of serological and molecular testing of positive subjects.

Bird Species	Gender	IFAT Titers	PCR Results
*Anas acuta*	F	50	B
*Anas acuta*	M	50	B
*Anas acuta*	F	50	B/H
*Anas crecca*	F	100	B/H
*Anas crecca*	F	50	B/H
*Anas crecca*	M	100	B
*Anas crecca*	F	50	B
*Anas crecca*	M	50	H
*Anas crecca*	M	50	neg
*Anas crecca*	F	100	neg
*Anas crecca*	F	50	neg
*Anas penelope*	M	50	B/H
*Anas penelope*	F	50	B
*Anas penelope*	F	50	neg
*Anas penelope*	F	50	neg
*Anas penelope*	M	50	neg
*Anas penelope*	F	50	neg
*Anas penelope*	M	50	neg
*Anas penelope*	M	50	neg
*Anas plathyrynchos*	F	50	B/H
*Anas plathyrynchos*	M	50	H
*Anas plathyrynchos*	F	50	BH
*Anas plathyrynchos*	M	50	H
*Anas plathyrynchos*	F	50	H
*Anas plathyrynchos*	F	50	B/H
*Anas plathyrynchos*	M	50	H
*Anas plathyrynchos*	M	50	H
*Anas plathyrynchos*	F	50	B
*Buteo buteo*	M	50	H
*Buteo buteo*	M	100	H
*Buteo buteo*	F	50	H
*Columba palumbus*	F	50	H
*Columba palumbus*	F	50	H
*Columba palumbus*	M	50	B/H
*Columba palumbus*	F	50	H
*Larus ridibundus*	M	100	neg
*Larus ridibundus*	F	100	H
*Larus ridibundus*	F	50	B
*Vanellus vanellus*	F	50	B
*Vanellus vanellus*	M	50	B
*Vanellus vanellus*	F	50	B/H

F = female, M = male, H = heart, B = brain, and neg = negative specimen

**Table 2:** Number, species, and gender of examined birds.

Bird Species	Males/Females	Total
Waterfowl		
*Anas acuta*	4/12	16
*Anas crecca*	31/41	72
*Anas penelope*	17/18	35
*Anas platyrhyncos*	19/22	41
*Anas clypeata*	5/6	11
*Vanellus vanellus*	3/4	7
Non-waterfowl		
*Asio otus*	3/0	3
*Athene noctua*	2/2	4
*Buteo buteo*	5/4	9
*Columba palumbus*	14/12	26
*Corvus frugilegus*	2/3	5
*Falco tinnunculus*	4/3	7
*Garrus glandarius*	3/1	4
*Larus ridibundus*	23/28	51
*Sturnus vulgaris*	2/2	4
*Sylvia melanocephala*	3/0	3
*Sylvia atricapilla*	1/3	4

## References

[1] Dubey J.P., Carpenter J.L., Speer C.A., Topper M.J., Uggla A. (1988). Newly recognized fatal protozoan disease of dogs. J. Am. Vet. Med. Assoc..

[2] Kobayashi Y., Yamada M., Omata Y., Koyama T., Saito A., Matsuda T., Okuyama K., Fujimoto S., Furuoka H., Matsui T. (2001). Naturally-occurring *Neospora caninum* infection in an adult sheep and her twin fetuses. J. Parasitol..

[3] Dubey J.P., Schares G., Ortega-Mora L.M. (2007). Epidemiology and control of neosporosis and *Neospora caninum*. Clin. Microbiol. Rev..

[4] Reichel M.P., Alejandra Ayanegui-Alcérreca M., Gondim L.F., Ellis J.T. (2013). What is the global economic impact of Neospora caninum in cattle - the billion dollar question. Int. J. Parasitol..

[5] Rossi G.F., Cabral D.D., Ribeiro D.P., Pajuaba A.C., Corrêa R.R., Moreira R.Q., Mineo T.W., Mineo J.R., Silva D.A. (2011). Evaluation of *Toxoplasma gondii* and *Neospora caninum* infections in sheep from Uberlândia, Minas Gerais State, Brazil, by different serological methods. Vet. Parasitol..

[6] de Barros L.D., Miura A.C., Minutti A.F., Vidotto O., Garcia J.L. (2018). *Neospora caninum* in birds: A review. Parasitol. Int..

[7] Tranas J., Heinzen R.A., Weiss L.M. (1999). Serological evidence of human infection with the protozoan *Neospora caninum*. Clin. Diagn. Lab. Immunol..

[8] de Oliveira U.V., de Magalhães V.C., Almeida C.P., Santos Idos A., Mota D.A., Macêdo L.S., Silva F.L., Carvalho F., dos S., Wenceslau A.A. (2013). Quails are resistant to infection with *Neospora caninum* tachyzoites. Vet. Parasitol..

[9] Oliveira S., Aizawa J., Soares H.S., Chiebao D.P., Castro M.B., Hora A.S., Lopes M.G., Schares G., Jenkins M.C., Kwok O.C.H. (2018). Experimental *Neospora caninum* infection in chickens (*Gallus gallus domesticus*) with oocysts and tachyzoites of two recent isolates reveals resistance to infection. Int. J. Parasitol..

[10] de Barros L.D., Taroda A., Miura A.C., Minutti A.F., Sasse J.P., Nino B.S.L., da Silva E.O., Headley S.A., Vidotto O., Garcia J.L. (2019). Experimental inoculation of Neospora caninum tachyzoites in eared doves (*Zenaida auriculata*). Exp. Parasitol..

[11] Gondim L.S., Abe-Sandes K., Uzêda R.S., Silva M.S., Santos S.L., Mota R.A., Vilela S.M., Gondim L.F. (2010). Toxoplasma gondii and Neospora caninum in sparrows (Passer domesticus) in the Northeast of Brazil. Vet. Parasitol..

[12] Darwich L., Cabezón O., Echeverria I., Pabón M., Marco I., Molina-López R., Alarcia-Alejos O., López-Gatius F., Lavín S., Almería S. (2012). Presence of *Toxoplasma gondii* and *Neospora caninum DNA* in the brain of wild birds. Vet. Parasitol..

[13] Salant H., Mazuz M.L., Savitsky I., Nasereddin A., Blinder E., Baneth G. (2015). Exposure to *Neospora* spp. and *Besnoitia* spp. in wildlife from Israel. Vet. Parasitol..

[14] Rocchigiani G., Poli A., Nardoni S., Papini R., Mancianti F. (2017). *Neospora caninum* in Wild Waterfowl: Occurrence of Parasite DNA and Low Antibody Titers. J. Parasitol..

[15] Mineo T.W., Carrasco A.O., Raso T.F., Werther K., Pinto A.A., Machado R.Z. (2011). Survey for natural *Neospora caninum* infection in wild and captive birds. Vet. Parasitol..

[16] Molina-López R., Cabezón O., Pabón M., Darwich L., Obón E., Lopez-Gatius F., Dubey J.P., Almería S. (2012). High seroprevalence of *Toxoplasma gondii* and *Neospora caninum* in the Common raven (*Corvus corax*) in the Northeast of Spain. Res. Vet. Sci..

[17] Lukášová R., Kobédová K., Halajian A., Bártová E., Murat J.B., Rampedi K.M., Luus-Powell W.J. (2018). Molecular detection of *Toxoplasma gondii* and *Neospora caninum* in birds from South Africa. Acta Trop..

[18] Nazir M.M., Ayaz M.M., Ahmed A.N., Maqbool A., Ashraf K., Oneeb M., Yasin G., Subhani A., Ali M.A., Nazir N. (2018). Prevalence of *Toxoplasma gondii*, *Neospora caninum*, and *Sarcocystis* Species *DNA* in the Heart and Breast Muscles of Rock Pigeons *(Columbia livia)*. J. Parasitol. Res..

[19] Müller N., Zimmermann V., Hentrich B., Gottstein B. (1996). Diagnosis of *Neospora caninum* and *Toxoplasma gondii* infection by PCR and *DNA* hybridization immunoassay. J. Clin. Microbiol..

